# Supplementary material for: The Role of Viral Dynamics and Infectivity in Models of Oncolytic Virotherapy for Tumours with Different Motility
Source: Bull Math Biol. 2026 Mar 25;88(5):66. doi: 10.1007/s11538-026-01630-6 (PMC13018097; doi:10.1007/s11538-026-01630-6)
Supplement: Supplementary file 1 — (pdf 135 KB) [file 11538_2026_1630_MOESM1_ESM.pdf]

Description of Electronic Supplementary Material for 'The role of viral dynamics and infectivity in models of oncolytic virotherapy for tumours with different motility'

David Morselli, Federico Frascoli, Marcello E. Delitala

Online Resource S1 (this file)

Description of the videos in the rest of the Electronic Supplementary Material.

Online Resource S2

Video to support Figs. 2 and 3a-b in the paper, showing the comparison in one spatial dimension between numerical simulations of the discrete models (solid lines), the numerical solution of the corresponding continuum models (dotted black lines) and the numerical solution of Eq. (U2) (dashed lines). The left panel refers to undirected cell movement and the right panel to pressure-driven cell movement. The parameters employed are the ones given in Table 1, with $\alpha = 580$ viruses/cells and $q_v = 1.67 \times 10^{-1}$ h^{-1}. The viral density of the agent-based model is multiplied by $q_v/(\alpha q)$ to allow the comparison with cell numbers; note that the viral densities for the continuous equations are not shown, as they would superimpose with infected cells. For the agent-based model, the densities of uninfected cells, infected cells and virus are represented respectively in blue, red and purple; the numerical solutions of Eqs. (2.2) and (2.3) are represented using the same colours. The vertical black dashed lines represent the expected positions of the uninfected fronts, travelling at speeds $2\sqrt{D_U p}$ and $\sqrt{D_P p/2}$. The horizontal solid black lines show the equilibrium of the ODE given by Eq. (2.9). The horizontal dashed yellow line represents the expected uninfected density at the front for Eq. (U2), given by Eq. (3.1), and the horizontal dash-dotted green line shows the analogue quantity in the case of Eq. (2.2). The results of the agent-based models are averaged over five simulations. The maximum of the cell density axes corresponds to the maximum over time of this average in the case of undirected movement.

Online Resource S3

Video to support Fig. 3c in the paper, showing the comparison in one spatial dimension between numerical simulations of the discrete models (solid lines), the numerical solution of the corresponding continuum models (dotted black lines) and the numerical solution of Eq. (U2) (dashed lines). The left panel refers to undirected cell movement and the right panel to pressure-driven cell movement. The parameters employed are the ones given in Table 1, with $\alpha = 3500$ viruses/cells and $q_v = 1$ h^{-1}. All the graphical elements have the same meaning as in the previous video S2.

Online Resource S4

Comparison in one spatial dimension between numerical simulations of the discrete model with pressure-driven cell movement (solid lines), the numerical solution of the corresponding continuum model (dotted black lines) and the numerical solution of Eq. (U3) (dashed lines). The parameters employed are the ones given in Table 1, with the exception of the diffusion coefficient of viral particles D_v (which is set to 10^{-5} mm^2/h). We used $\alpha = 580$ viruses/cells and $q_v = 1.67 \times 10^{-1}$ h^{-1} in the left panel; $\alpha = 3500$ viruses/cells and $q_v = 1$ h^{-1} in the right panel. The viral density of the agent-based model is multiplied by $q_v/(\alpha q)$ to allow the comparison with cell numbers; note that the viral densities for the continuous equations are not shown, as they would superimpose with infected cells. For the agent-based model, the densities of uninfected cells, infected cells and virus are represented respectively in blue, red and purple; the numerical solutions of Eq. (2.3) are represented using the same colours. The vertical black dashed lines represent the expected positions of the uninfected fronts, travelling at speed $\sqrt{D_P p/2}$. The horizontal solid black lines show the equilibrium of the ODE given by Eq. (2.9). The results of the agent-based models are averaged over five simulations. The maximum of the cell density axes corresponds to the maximum over time of this average in the case of undirected movement.

We remark that the simulations of the continuous models require a refined discretisation $\Delta x = 0.01$, $\Delta t = 10^{-4}$ to reduce the diffusion due to the numerical method.

Online Resource S5

Video to support Fig. 4c and the related discussion in the paper, showing the comparison in one spatial dimension between numerical simulations of the discrete model with pressure-driven cell movement (solid lines), the numerical solution of the corresponding continuum model (dotted black lines) and the numerical solution of Eq. (U3) (dashed lines). The parameters employed are the ones given in Table 1, with the with the exception of the diffusion coefficient of viral particles D_v (which is set to 10^{-5} mm^2/h) and the initial radius of viral infection R_v (which is set equal to R_u); as in the previous video, we here employ both combinations of α and q_v. All the graphical elements have the same meaning as in the previous video S4.

Online Resource S6

Video to support Fig. 5 in the paper, showing the comparison in two spatial dimensions between a single numerical simulations of the agent-based model with pressure-driven movement and the numerical solution of Eq. (2.3). The parameters employed are the ones given in Table 1, with the exception of the diffusion coefficient of viral particles D_v (which is set to 10^{-5} mm^2/h), the death rate of infected cells q (which is set to 8.33×10^{-3} h^{-1}, i.e. one-fifth of the reference values) and the initial radius of viral infection R_v (which is set equal to R_u); furthermore, we use the values $\alpha = 3500$ viruses/cells and $q_v = 1$ h^{-1}. The dashed cyan circles in the left panels represent the expected positions of the uninfected invasion fronts in the absence of treatment, travelling at speed $\sqrt{D_P p/2}$. The dashed red circles in the right panels represents the front of the infected cells given by the numerical solution of Eq. (U3), i.e., the circle of radius

$$R(t) = \sup \left\{ r \geq 0 \;\middle|\; i(t,r) \geq \frac{1}{\delta^2} \right\}$$

whenever the set is not empty. The maximum values of the colorbars correspond to the maximum over time of the quantity plotted.

Online Resource S7

Video to support Fig. 7 in the paper, showing the comparison in two spatial dimensions between a single numerical simulations of the agent-based model with undirected movement and the numerical solution of Eq. (2.2). The parameters employed are the ones given in Table 1, with the values $\alpha = 3500$ viruses/cells and $q_v = 1\ \mathrm{h}^{-1}$. The dashed cyan circles in the left panels represent the expected positions of the uninfected invasion fronts in the absence of treatment, travelling at speed $2\sqrt{D_U p}$. The dashed red circles in the right panels represents the front of the infected cells given by the numerical solution of Eq. (2.2), i.e., the circle of radius

$$R(t) = \sup\left\{ r \geq 0 \,\middle|\, i(t,r) \geq \frac{1}{\delta^2} \right\}$$

whenever the set is not empty. The maximum values of the colorbars correspond to K until time $t = 80$ and is then decreased to $K/4$ to enhance readability.

Online Resource S8

Video to support Fig. 8 in the paper, showing the comparison in two spatial dimensions between a single numerical simulations of the agent-based model with pressure-driven movement and the numerical solution of Eq. (2.3). The parameters employed are the ones given in Table 1, with the values $\alpha = 3500$ viruses/cells and $q_v = 1\ \mathrm{h}^{-1}$. The dashed cyan circles in the left panels represent the expected positions of the uninfected invasion fronts in the absence of treatment, travelling at speed $\sqrt{D_P p}/2$. The dash-dotted black circles represent the expected invasion speed in presence of virotherapy, given by Eq. (A.1) with $\bar{u} = 1800$ cells/mm^2; observe that in this second case we consider a wave starting from an initial radius of 3 mm rather than $R_u = 2.6$ mm, in order to compensate the transient behaviour in which the infection is far from the boundary of the tumour. The dashed red circles in the right panels represents the front of the infected cells given by the numerical solution of Eq. (2.2), i.e., the circle of radius

$$R(t) = \sup\left\{ r \geq 0 \,\middle|\, i(t,r) \geq \frac{1}{\delta^2} \right\}$$

whenever the set is not empty. The maximum values of the colorbars correspond to K until time $t = 80$ h and is then decreased to $K/4$ to enhance readability.
